# Anti-Thymocyte Globulin Treatment Augments 1,25-Dihydroxyvitamin D3 Serum Levels in Patients Undergoing Hematopoietic Stem Cell Transplantation

**DOI:** 10.3389/fimmu.2021.803726

**Published:** 2022-01-04

**Authors:** Carina Matos, Katrin Peter, Laura Weich, Alice Peuker, Gabriele Schoenhammer, Tobias Roider, Sakhila Ghimire, Nathalie Babl, Sonja Decking, Martina Güllstorf, Nicolaus Kröger, Kathrin Hammon, Wolfgang Herr, Klaus Stark, Iris M. Heid, Kathrin Renner, Ernst Holler, Marina Kreutz

**Affiliations:** ^1^ Department of Internal Medicine III, University Hospital Regensburg, Regensburg, Germany; ^2^ Department of Medicine V, University of Heidelberg, Heidelberg, Germany; ^3^ Regensburg Center for Interventional Immunology (RCI), Regensburg, Germany; ^4^ Department of Stem Cell Transplantation, University Medical Center Hamburg-Eppendorf, Hamburg, Germany; ^5^ Department for Genetic Epidemiology, University of Regensburg, Regensburg, Germany

**Keywords:** HSCT, vitamin D3, ATG, GvHD, dendritic cells

## Abstract

Application of anti-thymocyte globulin (ATG) is a widely used strategy for the prevention of graft-versus-host disease (GvHD). As vitamin D3 serum levels are also discussed to affect hematopoietic stem cell transplantation (HSCT) outcome and GvHD development, we analysed a possible interplay between ATG treatment and serum levels of 25-hydroxyvitamin D3 and 1,25-dihydroxyvitamin D3 in 4 HSCT cohorts with different vitamin D3 supplementation. ATG is significantly associated with higher serum level of 1,25-dihydroxyvitamin D3 around HSCT (day -2 to 7, peri-transplant), however only in patients with adequate levels of its precursor 25-hydroxyvitamin D3. ATG exposure had no impact on overall survival in patients supplemented with high dose vitamin D3, but was associated with higher risk of one-year treatment-related mortality (log rank test p=0.041) in patients with no/low vitamin D3 supplementation. However, the difference failed to reach significance applying a Cox-model regression without and with adjustment for baseline risk factors (unadjusted P=0,058, adjusted p=0,139). To shed some light on underlying mechanisms, we investigated the impact of ATG on 1,25-Dihydroxyvitamin D3 production by human dendritic cells (DCs) *in vitro.* ATG increased gene expression of *CYP27B1*, the enzyme responsible for the conversion of 25-hydroxyvitamin D3 into 1,25-dihydroxyvitamin D3, which was accompanied by higher 1,25-dihydroxyvitamin D3 levels in ATG-treated DC culture supernatants. Our data demonstrate a cooperative effect of 25-hydroxyvitamin D3 and ATG in the regulation of 1,25-dihydroxyvitamin D3 production. This finding may be of importance in the context of HSCT, where early high levels of 1,25-dihydroxyvitamin D3 levels have been shown to be predictive for lower transplant related mortality and suggest that vitamin D3 supplementation may especially be important in patients receiving ATG for GvHD prophylaxis.

## Introduction

Allogeneic hematopoietic stem cell transplantation (aHSCT) is a potentially curative treatment modality for different haematological malignancies, however its success is limited due to associated complications such as infections or graft- versus- host disease (GvHD) which still remain the major cause of non-relapse mortality in aHSCT (1).

Anti-thymocyte globulin (ATG), a polyclonal antibody-mixture raised in rabbits against the human lymphoblastic T cell line Jurkat, is an immunosuppressive drug used for GvHD prophylaxis during conditioning. A meta-analysis reported that ATG use is associated with reduced risk of acute and chronic GVHD. However, the efficacy of ATG in the prevention of GvHD in HSCT patients may depend on many confounding variables such as dose, type and timing of its administration and transplantation characteristics (2). Administration of ATG results in T-cell depletion, which is presumed to represent the main mechanism by which ATG reduces the incidence of GvHD (3).

Vitamin D3 insufficiency, usually defined as serum levels of its stable metabolite 25-hydroxyvitamin D3, is a common finding (not only) in HSCT patients, however, the clinical impact of vitamin D3 deficiency is controversially discussed. A recent meta-analysis described no statistically significant association between 25-hydroxyvitamin D3 deficiency and neither acute nor chronic GVHD (4). In contrast, Radujkovic and colleagues showed in two cohorts (n=890 patients) that pre-transplant 25-hydroxyvitamin D3 deficiency (<20 ng/mL) was associated with a higher risk of relapse in patients allografted for myeloid malignancies (5).

Although the impact of 25-hydroxyvitamin D3 is extensively studied in the context of HSCT and GvHD, little is known about 1,25-dihydroxyvitamin D3, the active form of vitamin D3. It is textbook knowledge that 1,25-dihydroxyvitamin D3 is produced in the kidney, while its precursor vitamin D3 is present in high amounts in the skin or in the gut, where it is taken up from the diet. Both organs represent immunological ‘‘barriers’’ and target organs for GvHD development and local extrarenal production of 1,25-dihydroxyvitamin D3 might be of importance for immune regulation. We previously showed that myeloid cells such as dendritic cells (DC) express the vitamin D3-1–hydroxylase CYP27B1 and thus are able to convert 25-hydroxyvitamin D3 to bioactive 1,25-dihydroxyvitamin D3 (6–8) which may support local immunosuppression in skin and gut (9).

Analysing a discovery cohort consisting of 143 HSCT patients, our data highlight peri-transplant (day −2 to 7), 1,25-dihydroxyvitamin D3 levels, but not the commonly monitored 25-hydroxyvitamin D3 levels, as potent predictor of 1-year transplant-related mortality (TRM). This finding was further confirmed by analysing three additional cohorts, consisting altogether of 365 patients and suggest to monitor both vitamin D3 metabolites in HSCT patients (10).

Cyclosporine, Dexamethasone and ATG are known immunosuppressive treatments used for GvHD prophylaxis. Here we analysed whether these typical transplant-related drugs influence 1,25-dihydroxyvitamin D3 production by monocyte- derived dendritic cells (DCs) *in vitro*. Our data indicate that besides its classical role for T cell depletion, ATG may also impact the immune response in patients *via* modulation of the vitamin D3 metabolism.

## Material and Methods

### Patient Characteristics

Four cohorts with a total of n=508 patients were included in our analyses. The discovery cohort consisted of n=143 patients at the Regensburg University Medical Center with HSCT between May 2012 and February 2015. All HSCT recipients in the discovery cohort received oral high dose vitamin D3 supplementation (Vigantol oil, 20.000 IU/ml, Merck) consisting of a 50,000 IU-dose upon admission to hospital (d-16 to d-6) followed by daily administration of 10,000 IU. To monitor 25-hydroxyvitamin D3 and 1,25-dihydroxyvitamin D3 serum levels, blood was drawn repeatedly during inpatient stay, and thereafter during routine outpatient visits. Measurements were performed at least once during the indicated time intervals. When multiple measurements were available for the same time interval, the median value was used. Serum calcium levels were assessed twice a week. The described supplementation dose was maintained until patients reached 25-hydroxyvitamin-D3 serum levels of 150–200 nmol/L with subsequent dose adjustment to avoid 25-hydroxyvitamin-D3 levels >150–200 nmol/L

Our replication stage consisted of three patient cohorts from various clinical settings to replicate our initial findings and to generalize for other clinical settings: (I) HSCT patients from Regensburg transplanted between March 2015 and May 2017 receiving the same high-dose vitamin D3 supplementation as the discovery cohort, (II) HSCT patients from Regensburg transplanted between March 2011 and February 2013 receiving vitamin D3 supplementation at lower dose (ranging from 1000 to 5000 IU/d, Vigantoletten, 1000 IU/tablet, Merck), (III) HSCT patients from the University Medical Center Hamburg-Eppendorf transplanted between February 2012 and August 2014 receiving no vitamin D3 supplementation. Eligibility and exclusion criteria for all three replication groups were the same as in the discovery cohort, yielding n=115, n=107 and n=143 patients in replication cohort I, II, and III, respectively. All cohorts analysed in the present study were already described in detail in (10).

### Isolation of Monocytes

Monocytes were isolated with the approval of local ethic committee, from healthy donors as described previously (11). All human participants gave written informed consent.

### Culture of Monocyte-Derived DCs

For DC differentiation, 0.5 to 1.0 × 10^6^ monocytes/mL were cultured for five days in RPMI medium supplemented with 10% fetal calf serum (PAN Biotech), IL-4 (144 U/mL), and granulocyte macrophage colony-stimulating factor (GM-CSF, 225 U/mL; both from PeproTech, Hamburg, Germany). iDCs were then stimulated with 100 ng/mL LPS (from Salmonella abortus equi S-form, Enzo Life Sciences, Lörrach, Germany), 25-hydroxyvitamin D3 (Sigma-Aldrich) (25 nM to 100 nM) and or ATG (Fresenius, Bad Homburg, Germany) (now named Grafalon^®^, distributed by Neovii Biotech, Gräfelfing, Germany) (100 µg/mL), Cyclosporine A (Sandimmun, Novartis), Dexamethasone (Jenapharm, mibe GmbH), IgG isotype control (polyclonal, rabbit, Molecular Innovations, Novi, MI, USA) (100 µg/mL) for 48 hours.

### Preparation of RNA, Reverse Transcription, and Quantitative Real-Time PCR

Total cellular RNA was extracted using the RNeasy Mini Kit (Qiagen, Hilden, Germany). RNA concentration was measured using a NanoDrop Spectrophotometer (Thermo Fisher Scientific, Schwerte, Germany). Reverse transcription was performed with 500 ng RNA in a total volume of 20 μl using an M-MLV Reverse Transcriptase from Promega (Mannheim, Germany). For reverse transcription-quantitative real-time PCR, 1 μl cDNA, 0.5 μl of primers (10 μM), and 5 μl QuantiFast SYBR Green PCR Kit (Qiagen) in a total of 10 μl were applied, using the Mastercycler Ep Realplex (Eppendorf). Primer sequences (all from Eurofins MWG Operon, Ebersberg, Germany) were as follows (-5´-3´); (F- Forward; R- Reverse): CYP27A1_F: GTCTGGCTACCTGCACTTCTTACTG CYP27A1_R: TCAGGGTCCTTTGAGAGGTGGT CYP27B1_F: TGGCAGAGCTTGAATTGCAAATGG; CYP27B1_R: ACTGTAGGTTGATGCTCCTTTCAGGT; 18S_F: ACCGATTGGATGGTTTAGTGAG; 18S_R: CCTACGGAAACCTTGTTACGAC

### Preparation of Whole Cell Lysates and Western Blotting

Whole cell lysates were prepared using RIPA buffer (Sigma-Aldrich) and quantified with the Qubit Protein Assay Kit (Thermo Fisher Scientific). Samples were separated by 12% SDS-PAGE and transferred to PVDF membranes, blocked with 5% milk (Sucofin) in TBS buffer with 0.1% Tween for 1 h, and incubated with primary antibodies overnight: anti-VDR ((D2K6W) Cell Signaling Technology, Danvers, MA, USA, clone or anti-actin (Sigma Aldrich). Membranes were incubated with secondary antibodies for 1 h at RT and analyzed using the chemiluminescence system Fusion Pulse 6 (Vilber Lourmat).

### Vitamin D Measurement

Vitamin D levels were measured directly after serum withdrawal or from sera stored at -80°C by the Department of Clinical Chemistry, University Medical Center of Regensburg. From May 2012 to October 2014, 25-hydroxyvitamin D3 serum levels were analysed by a chemiluminescence immunoassay according to the manufacturer’s instructions (Immunodiagnostic systems, Frankfurt am Main, Germany). After attesting for comparability, from November 2014 on, 25-hydroxyvitamin D3 serum levels were analyzed by liquid chromatography high-resolution tandem mass spectrometry as described in (12). 1,25-dihydroxyvitamin D3concentrations were measured using a radioimmunoassay according to the manufacturer’s instructions (Immunodiagnostic systems, Frankfurt am Main, Germany) by the Department of Clinical Chemistry, University Medical Center of Regensburg. For the replication cohorts and DC supernatants, 1,25-dihydroxyvitamin D3levels were measured by the MVZ Laborzentrum Ettlingen, Germany, using the same method as described above.

### Statistical Analysis

Statistics were calculated using GraphPad Prism, Version 8 (La Jolla, CA, USA) or using SPSS Statistics version 26 (IBM, Armonk, USA). Comparisons between groups were performed using the appropriate statistical methods depending on Gaussian distributions, number of groups and variables. A value of p < 0.05 was considered statistically significant. To examine one-year-survival, a Kaplan-Meier curve was generated to visualize differences between patients receiving ATG with patients that did not receive ATG. The log rank test and an unadjusted Cox-model were used to test for difference in survival of patients.

## Results

### Higher 1,25-Dihydroxyvitamin D3 but Not 25-Hydroxyvitamin D3 Serum Levels in Patients With ATG Therapy

**Figure 1:**
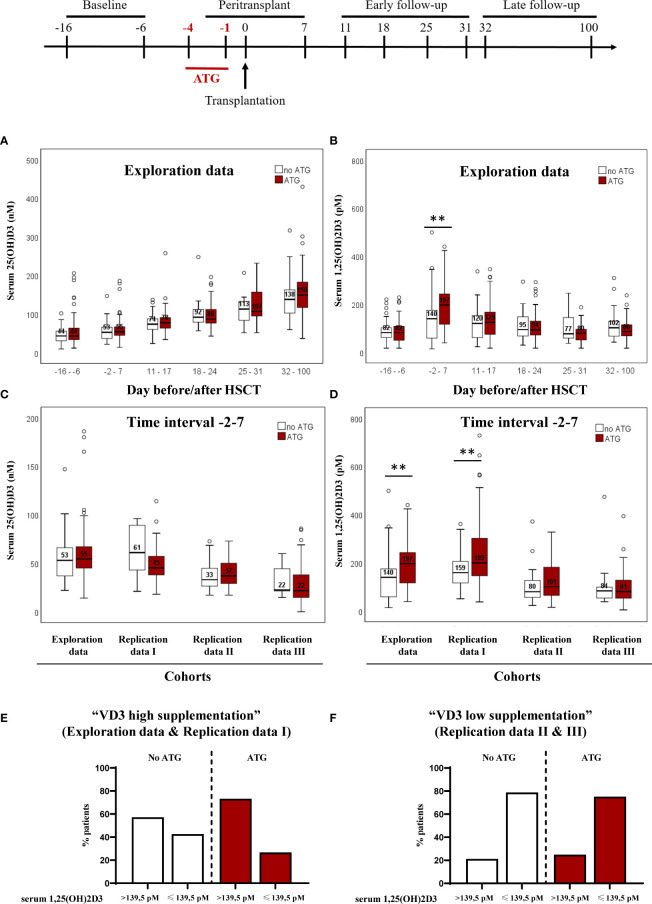
Impact of ATG treatment on 25(OH)D3 and 1,25(OH)2D3 circulating serum levels of patients undergoing hematopoietic stem cell transplantation Panel **(A)** depicts the median serum 25-hydroxyvitamin D3 levels followed over time in association with ATG treatment for the discovery cohort. In panel **(B)**, the median serum 1,25(OH)2D3 level is presented. Panel **(C)** depicts the median serum 25(OH)D3 levels from days -2 to 7 around HSCT of patients receiving ATG and patients that did not received ATG. In panel **(D)**, the median serum 1,25(OH)2D3 level is presented. Numbers indicate median serum 25(OH)D3 and 1,25(OH)2D3 values, error bars indicate 95% confidence interval. Statistical analysis was performed with Mann-Whitney-U test, two-tailed (**p < 0.01). In **(E, F)** the distribution of patients below and above the calculated cut-off is shown, in relation to ATG treatment for the cohorts with high and low/no vitamin D3 supplementation, respectively.

Vitamin D3 metabolites were already shown to be implicated in the outcome of HSCT. Although the impact of 25-hydroxyvitamin D3 was debated in several studies (13, 14), the active metabolite 1,25-dihydroxyvitamin D3 did not gather the same attention. In a recent study, our group demonstrated that high 1,25-dihydroxyvitamin D3 levels are predictive for treatment related mortality. Using the same cohorts of patients and in order to understand the factors that influence vitamin D3 levels, we evaluated the influence of typical transplant-related factors on 1,25-dihydroxyvitamin D3 and 25-hydroxyvitamin D3 levels. The discovery cohort consisted of n=143 patients at the Regensburg University Medical Center and received oral high-dose vitamin D3 supplementation. The replication cohort I consisted of patients with oral high-dose vitamin D3 supplementation similar to the discovery cohort. The other two replication cohorts received either vitamin D3 supplementation at lower dose, or no vitamin D3 supplementation. 25-hydroxyvitamin D3 levels were followed over time and are presented in association with ATG treatment (**Figure 1A**). 25-hydroxyvitamin D3 levels increased over time but no impact of ATG administration was observed. When analyzing 1,25-dihydroxyvitamin D3 levels (**Figure 1B**) we observed that ATG administration led to higher 1,25-dihydroxyvitamin D3 in the time frame around transplantation (days -2 to 7). We subsequently analyzed the effect of ATG administration in our three additional cohorts (Replication cohorts I, II and III) for the time interval around transplantation (days -2 to 7).

ATG application showed no association with 25-hydroxyvitamin D3 levels measured at peri-transplant time (**Figure 1C**) for all of the 4 cohorts. Although no impact of ATG administration was observed, basal and ATG-stimulated 25-hydroxyvitamin D3 levels differed between the cohorts and only the exploration cohort and the replication cohort I presented 25-hydroxyvitamin D3 levels higher than 50 nM, a concentration usually considered adequate. In contrast to 25-hydroxyvitamin D3, patients receiving ATG therapy revealed higher 1,25-dihydroxyvitamin D3 compared with patients that did not receive ATG (**Figure 1D**) in the exploration cohort and the replication cohort I, where basal 25-hydroxyvitamin D3 levels met the criteria for vitamin D sufficiency [see Peter et al. (10)].

Our previous study highlighted peritransplant 1,25-dihydroxyvitamin D3levels, but not the commonly monitored 25-hydroxyvitamin D3 levels, as potent predictor of 1-year transplant-related mortality (TRM) independent of severe aGvHD. The optimal threshold for 1,25-dihydroxyvitamin D3 to identify patients at risk was 139.5 pM (10). Next we analyzed patient distribution below and above this cut-off in relation to ATG administration (**Figures 1E, F**). In the two cohorts (exploration combined with replication I) with high vitamin D3 supplementation, 73,3% of ATG-treated patients had 1,25-dihydroxyvitamin D3 levels above the cut-off. Without ATG treatment 57,3% showed 1,25 levels above the threshold (**Figure 1E**). In contrast, patients with low/no supplementation (replication II combined with replication III), only 24,9% with ATG and 21,2% without ATG treatment reached higher levels than the defined cut-off (**Figure 1F**). These results demonstrate the importance of sufficient 25-hydroxyvitamin D3 serum levels for the described positive effect of ATG on 1,25-dihydroxyvitamin D3 production.

### ATG Treatment Increases 1,25-Dihydroxyvitamin D3 Production by Human Monocyte-Derived Dendritic Cells (DCs) *In Vitro*

**Figure 2:**
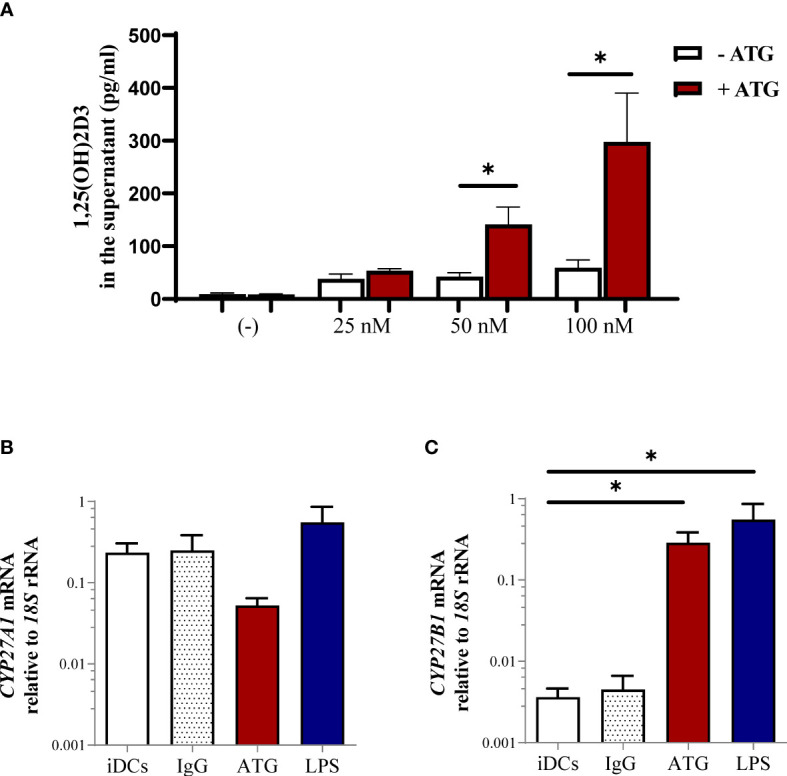
Impact of ATG treatment on production of 1,25(OH)2D3 by human monocyte-derived dendritic cells (DCs). **(A)** Human monocyte-derived DCs were stimulated for 24 h in the presence of different 25(OH)D3 concentrations in the presence or absence of ATG (100 µg/ml). After 24 h the production of 1,25(OH)2D3 was analyzed by means of chemiluminescence immunoassay. Data are means ± SEM (n≥ 3). Statistical analysis was performed using Mann-Whitney-U test, two-tailed. Panel **(B)** depicts CYP27A1 and **(C)** shows CYP27B1 mRNA expression of DCs analyzed by means of quantitative real-time PCR relative to 18S rRNA expression. Data are means ± SEM (n = 4). Statistical analysis was performed using Kruskal-Wallis and Dunn’s posthoc test [*p ≤ 0.05, tested versus immature DC (control)].

To shed some light on the interplay between ATG administration and 1,25-dihydroxyvitamin D3 production and to confirm a dependence on sufficient 25-hydroxyvitamin D3 levels, we incubated human monocyte-derived dendritic cells with ATG in the presence of different concentrations of the vitamin D3 precursor 25-hydroxyvitamin D3. As observed in **Figure 2A**, spontaneous conversion of 25-hydroxyvitamin D3 into 1,25-dihydroxyvitamin D3 was very low and not dependent on the provided 25-hydroxyvitamin D3 level in the culture medium. However, ATG treatment of monocyte-derived DCs increased the conversion of 25-hydroxyvitamin D3 to 1,25-dihydroxyvitamin D3 but only when DCs were cultured in the presence of 25-hydroxyvitamin D3 precursor levels in concentrations superior to 50 nM.

To investigate the possible mechanism by which ATG leads to the enhanced production of the active metabolite 1,25-dihydroxyvitamin D3, we incubated human monocyte-derived DCs with ATG-Fresenius and analyzed enzymes related to vitamin D3 metabolism. CYP27A1, the enzyme involved in the production of 25-hydroxyvitamin D3 from vitamin D3 and CYP27B1, the enzyme converting 25-hydroxyvitamin D3 to the active 1,25-dihydroxyvitamin D3 metabolite. We detected a trend towards a downregulation of *CYP27A1* mRNA upon treatment with ATG, indicating that ATG could potentially also modulate the first step of vitamin D3 metabolism (**Figure 2B**). In contrast, the expression of *CYP27B1* (**Figure 2C**) was significantly upregulated by ATG treatment. Lipopolysaccharide was used as a positive control and led to a comparable induction of *CYP27B1* mRNA (**Figure 2C**). Accordingly, LPS and ATG incubation induced comparable amounts of 1,25-dihydroxyvitamin D3 in supernatants of DC cultures (data not shown). This would be in line with a causal model that ATG therapy stimulates directly 1,25-dihydroxyvitamin D3 production in (myeloid) cells and in turn leads to increased 1,25-dihydroxyvitamin D3 serum levels after ATG administration.

### ATG but Not Other Immunosuppressive Drugs Such as Cyclosporine or Dexamethasone Induce 1,25-Dihydroxyvitamin D3 Production and VDR Expression in DCs

**Figure 3:**
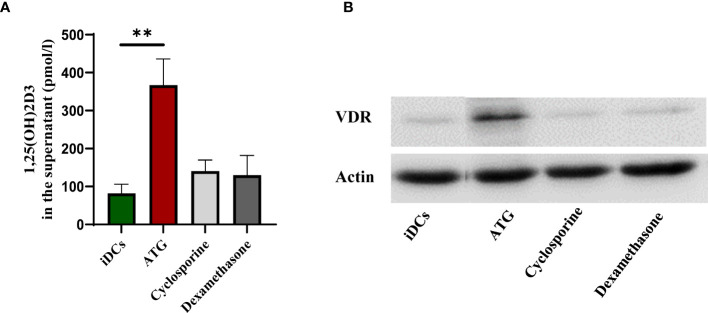
Comparative effect of ATG, Cyclosporine and Dexamethasone on 1,25(OH)2D3 production and VDR expression. Human monocyte-derived DCs were stimulated for 24 h in the presence 100 nM 25(OH)D3 with or without ATG (100 µg/ml), Cyclosporine A (1,7 µM) and Dexamethasone (100 nM). Panel **(A)** After 24 h the production of 1,25(OH)2D3 was analyzed by means of chemiluminescence immunoassay. Data are means ± SEM (n = 3). Statistical analysis was performed using Kruskal-Wallis and Dunn’s posthoc test [**p ≤ 0.01, tested versus immature DC (control)]. In **(B)**, VDR expression was analysed by western blot. A representative donor is shown.

Due to our observation that ATG treatment upregulates the expression of *CYP27B1* resulting in higher 1,25-dihydroxyvitamin D3 production, we sought to investigate whether other immunosuppressive drugs such as cyclosporine and dexamethasone would have the same effect. Cyclosporine A, a calcineurin inhibitor, is a key immunosuppressive drug administered in the transplantation setting and it has been demonstrated that cyclosporine increases 1,25-dihydroxyvitamin D3 levels in rats (15). The interplay between Dexamethasone, and 1,25-dihydroxyvitamin D3 was already demonstrated in different studies (16–18). Hidalgo et al. (16) demonstrated the synergism between 1,25-dihydroxyvitamin D3 and dexamethasone in inhibiting cell growth and increasing vitamin D receptor (VDR) expression. Therefore, we incubated human monocyte-derived DCs with 25-hydroxyvitamin D3combined with either ATG, dexamethasone or cyclosporine. As observed in **Figure 3A**, only ATG treatment led to a significant increase in 1,25-dihydroxyvitamin D3 production by DCs. Interestingly, the expression of the VDR (**Figure 3B**) was also upregulated in myeloid cells after ATG treatment which could probably lead to a paracrine immune suppression of DCs by the produced 1,25-dihydroxyvitamin D3.

### ATG Induces a Tolerogenic Phenotype in DCs

**Figure 4:**
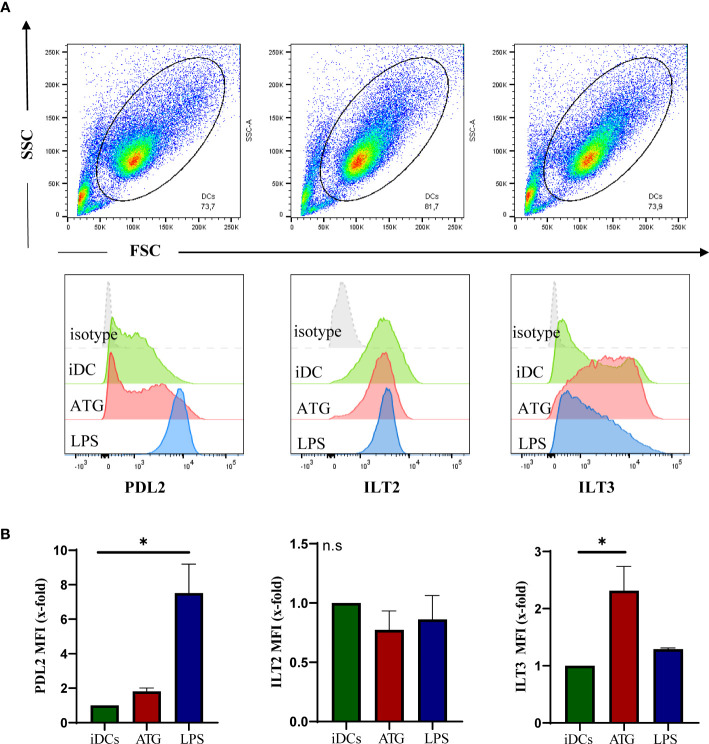
ATG effect on DC phenotype. Human monocyte-derived DCs were stimulated for 48 h with or without ATG (100 µg/ml) or LPS. Afterwards, cells were harvested, washed and stained by means of flow cytometry. Shown is a representative dot blot of the respective cell populations and overlaid histograms of the isotype (grey) **(A)**. Median fluorescence intensities are summarized in **(B)**. Data are means ± SEM (n = 3). Statistical analysis was performed using Kruskal-Wallis posthoc test (*p ≤ 0.05, tested versus immature DC (control); n.s. not significant).

Tolerogenic dendritic cells are characterized by the expression of co-inhibitory molecules such as Ig-like transcripts (ILTs), low expression of costimulatory molecules (e.g. CD83, CD86) and no or little production of proinflammatory cytokines such as IL-12 or IL-6 (19, 20). As 1,25-dihydroxyvitamin D3 is known to induce tolerogenic DCs (21), we next analyzed co-inhibitory molecules on DCs after exposure to ATG. Surface expression of ILT-3 but not ILT-2 or PD-L2 was significantly upregulated in the presence of ATG indicating that paracrine or autocrine 1,25-dihydroxyvitamin D3 production may support a more tolerogenic phenotype of DC (**Figures 4A, B**). In line with our previous work (22), ATG also induced a semi-mature phenotype with reduced expression of CD80, CD83 and CD86 in comparison to LPS and did not induce IL-12 nor IL-6 secretion (data not shown) (22). Combined application of 25-hydroxyvitamin D3 and ATG or did not alter cytokine secretion. In addition, no significant amounts of IL-12 or IL-6 were detected after cyclosporine and dexamethasone treatment (data not shown).

### ATG Has No Impact on Patients Survival Supplemented With High Dose Vitamin D3

**Figure 5:**
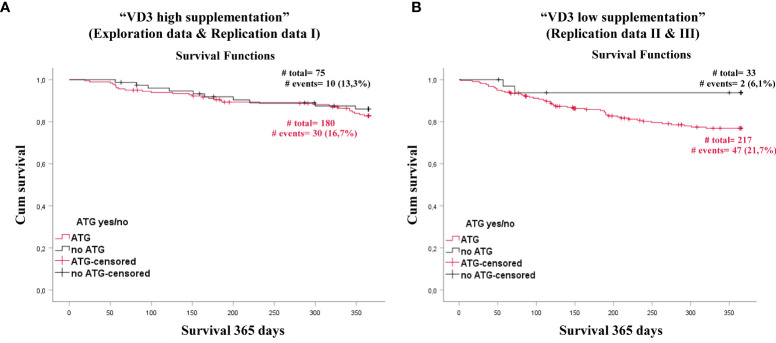
ATG treatment impact on treatment related survival on vitamin D high or vitamin D low supplemented patients. Shown is a Kaplan- Meier curve comparing patients that received ATG (red) with patients that did not received ATG treatment (black) for patients receiving high vitamin D3 supplementation **(A)** and patients receiving no or low vitamin D3 supplementation **(B)**.

To clarify a potential impact of interplay between vitamin D3 supplementation and ATG we performed survival analyses. In a combined analysis of patients from our discovery cohort and replication cohort I (n= 255, all with high dose vitamin D3 supplementation) ATG had no impact on patient survival (**Figure 5A**). Surprisingly, in patients with low/no vitamin D3 supplementation (n= 250, combined replication cohort II and III), the ATG exposure group had a diminished survival compared to patients without ATG treatment (Log Rank analysis p=0.041) (**Figure 5B**). Conversely, after applying a Cox-model regression without and with adjustment for baseline risk factors such as sex and age, the difference failed to reach significance (unadjusted p=0.058, adjusted p=0.139). As shown in **Table 1**, the only factor that remained significant after adjustment was the age of the patients. Based on these data, we suggest that especially patients treated with ATG should be supplemented with high dose vitamin D3.

**Table 1:** Association between ATG treatment, high/low vitamin D3 supplementation and TRM.

Model		Cox Regression				
	High vitamin D supplementation n = 255			Low vitamin D supplementation n = 250		
	#at risk/TRM	Exp(B)/HR (95% CI)	P value	#at risk/TRM	Exp(B)/HR (95% CI)	P value
**Unadjusted**	255/40			250/49		
ATG yes/no		1.253 (0.612; 2.563)	0.537		3.929 (0.954; 16.181)	0.058
**Adjusted I**	255/40			250/49		
ATG yes/no		1.136 (0.555; 2.325)	0.728		2.953 (0.703; 12.412)	0.139
age		1.096 (1.047; 1.147)	**0.000078**		1.033 (1.007; 1.059)	**0.012**
**Adjusted II**	255/40			250/49		
ATG yes/no		1.074 (0.522; 2.212)	0.846		2.956 (0.705; 12.398)	0.138
age		1.096 (1.048; 1.147)	**0.000064**		1.032 (1.007; 1.059)	**0.013**
sex		1.531 (0.819; 2.860)	0.182		0.734 (0.389; 1.385)	0.340
**Adjusted III**	235/38			250/49		
ATG yes/no		1.032 (0.496; 2.148)	0.932		3.012 (0.718; 12.635)	0.132
age		1.099 (1.047; 1.153)	**0.000120**		1.034 (1.006; 1.062)	**0.015**
sex		1.640 (0.863; 3.120)	0.131		0.718 (0.379; 1.359)	0.309
tumor stage		0.959 (0.500; 1.841)	0.900		0.831 (0.461; 1.496)	0.537
conditioning		2.091 (0.499; 8.769)	0.313		0.815 (0.364; 1.824)	0.619

Shown are the results from Cox proportional hazard models for the association between ATG treatment in patients with high or low vitamin D supplementation with TRM without and with adjustment for risk factors. P values ≤0,05 are marked in bold.

## Discussion

Although several studies addressed the importance of 25-hydroxyvitamin D3 serum levels in the context of HSCT, little is known about the active 1,25-dihydroxyvitamin D3 metabolite. We previously demonstrated that peri-transplant 1,25-dihydroxyvitamin D3 levels were the only significant independent predictor for one-year-survival besides severe aGvHD (10).

Nevertheless, why patients with high 1,25-dihydroxyvitamin-D3 levels are at less risk for TRM than those with low levels remains unknown. It is possible that high 1,25-dihydroxyvitamin D3 levels around transplantation result in an immunosuppressive environment as 1,25-dihydroxyvitamin D3 has been shown e.g. to tolerize dendritic cells and induce regulatory T cells (23–25). Similar results have been reported for ATG (22). In light of our results, one could speculate that part of ATG immune regulatory effect is based on the induction of 1,25-dihydroxyvitamin D3, as patients with ATG had a peak of 1,25-dihydroxyvitamin-D3 around transplantation.

Our *in vitro* experiments demonstrate the importance of adequate 25-hydroxyvitamin-D3 levels for the ATG effect. In concentrations below 50 nM, no positive effect on 1,25-dihydroxyvitamin D3 production was observed upon ATG treatment. This result goes in line with the observation made in patients: 1,25-dihydroxyvitamin D3 levels were only enhanced by ATG treatment in the discovery and replication cohort 1. These were the cohorts where the 25-hydroxyvitamin D3 serum levels met the sufficiency criteria (above 50 nM).

A more detailed *in vitro* analysis demonstrated that ATG treatment of human monocyte-derived dendritic cells lead to an upregulation of *CYP27B1* at mRNA level and resulted in a higher 1,25-dihydroxyvitamin D3 amount in culture supernatants. Since this enzyme is responsible for the conversion of 25-hydroxyvitamin-D3 to 1,25-dihydroxyvitamin D3, this result provides a possible mechanism by which ATG could be involved in 1,25-dihydroxyvitamin-D3 synthesis. As other immunosuppressive drugs such as cyclosporine and dexamethasone had no effect on 1,25-dihydroxyvitamin D3 production, the ATG effect seems to be specific and not related to its immunosuppressive activity. However, a study by Lee et al. described that cyclosporine treatment can also result in increased 1,25-dihydroxyvitamin D3 serum levels in mice (26), accompanied by higher renal CYP27B1 expression and decreased VDR expression. These results are not contradictory to ours as 1,25-dihydroxyvitamin-D3 levels seem to depend on classical renal production of 1,25-dihydroxyvitamin D3 in this study, whereas our *in vitro* data with human cyclosporine-treated DCs indicate that extrarenal production of 1,25-dihydroxyvitamin D3 by myeloid cells is not regulated by cyclosporine. Further studies in patients should clarify the role of cyclosporine on 1,25-dihydroxyvitamin D3 production *in vivo*.

Dexamethasone is a glucocorticoid drug used to treat a number of conditions such as rheumatic problems, allergies and asthma (27, 28). The synergistic effect between dexamethasone and Vitamin D3 was already demonstrated in several studies (16, 29–31). In our hands, dexamethasone did not increase 1,25-dihydroxyvitamin D3 levels nor modify the VDR expression in human DCs. In a study conducted by Akeno et al. (32) the authors investigated the effects of dexamethasone on CYP27B1 and CYP24A1. In line with our data, they did not detect an increase in 1,25-dihydroxyvitamin D3 levels in mice fed either a normal diet nor in mice fed a calcium and vitamin D3- deficient diet.

Hidalgo et al. (16) also studied the interaction between 1,25-dihydroxyvitamin D3 and dexamethasone. The authors found that treatment of murine squamous cell carcinoma cells with dexamethasone in combination with 1,25-dihydroxyvitamin D3 lead to the upregulation of VDR. In contrast, dexamethasone alone was not able to upregulate VDR expression. In our setting, dexamethasone was used in combination with 25-hydroxyvitamin D3, the precursor of 1,25-dihydroxyvitamin D3. Although human DCs express CYP27B1 and have the capacity to produce 1,25-dihydroxyvitamin D3 in a autocrine fashion, it can well be that the amount of 1,25-dihydroxyvitamin-D3 produced by the cells was not enough to generate a sufficient level to synergize with dexamethasone and upregulate the VDR. Taken together, our data provide a possible mechanism by which ATG leads to an increased 1,25-dihydroxyvitamin D3 serum levels in patients with sufficient 25-hydroxyvitamin D3. Together, both compounds can synergize in creating a “tolerogenic” environment that may help to maintain the immune balance.

ATG is normally used in patients receiving a graft from unrelated donors (33) but Kröger et al. (34) demonstrated that ATG-treatment can also significantly lower the incidence of chronic GvHD (cGvHD) after allogeneic transplantation from HLA-identical siblings. Admiraal et al. showed that optimum ATG dosing is associated with higher survival probability (35).

There are different ATG preparations, raised either in horses or rabbit by immunization with human thymocytes or with Jurkat T-cell line (36). The antigens targeted by the different preparations have been well described and differ in specificity. While rabbit ATG (thymoglobulin, (THG) also known as ATG-Genzyme, Sanofi Genzyme) is derived from rabbits immunized with human thymocytes, ATG Fresenius is produced in rabbits immunized with the Jurkat T-cell line (37). It has been demonstrated that although THG contains antibodies against several T- and B- cell antigens such as CD2, CD3, CD4, CD8, CD11, CD20, CD25, human leukocyte antigen DR (HLA-DR) and HLA class I, ATG-Fresenius is infrequently active against CD3, CD4 and HLA-DR but instead targets CD28, CD29, CD45, CD98 and CD147. Furthermore, competitive binding assays have demonstrated that THG has stronger reactivity than ATG-Fresenius to activated peripheral blood mononuclear cells. Based on this, the doses of ATG given depend on the chosen ATG preparation and are typically higher for ATG-Fresenius than for THG. Consequently, is seems safe to assume that ATG-Fresenius and THG differ in their immunosuppressive activity. Whether the impact on vitamin D3 metabolism depends on the given ATG preparation remains unclear and more studies should be conducted to clarify this interesting aspect.

In our analysis, we did not find any significant association of ATG with survival after applying Cox analyses. This could be due to the fact that our analysis incorporated the use of ATG in a dichotomous fashion and not in a concentration-dependent manner. However, it has to be emphasized that not all patients receiving ATG had high 1,25-dihydroxyvitamin D3 levels and that high levels also occurred in patients without ATG treatment. Therefore, other factors are obviously involved in the regulation of peri-transplant 1,25-dihydroxyvitamin D3 levels besides ATG and sufficient serum 25-hydroxyvitamin D3 levels that allow conversion to 1,25-dihydroxyvitamin D3.

Further studies should identify other factors that determine early 1,25-dihydroxyvitamin D3 serum levels in HSCT patients. Alternatively, supplementation with 1,25-dihydroxyvitamin D3 analogs could be an option to increase early 1,25-dihydroxyvitamin D3 levels to support patient survival.

## Data Availability Statement

The original contributions presented in the study are included in the article/supplementary materials, further inquiries can be directed to the corresponding author.

## Ethics Statement

The studies involving human participants were reviewed and approved by Ethics Committee University Hospital Regensburg. The patients/participants provided their written informed consent to participate in this study.

## Author Contributions

Conceptualization, KP and MK. Methodology, CM, AP, GS, and LW. Investigation, CM, NB, SD, SG, and KH. Writing—original draft preparation, CM. Writing—review and editing, MK, KR, IH, KS, WH, and EH. Supervision, MK. Funding acquisition, MK and KP. All authors have read and agreed to the published version of the manuscript.

## Funding

This research was funded by the Deutsche Forschungsgemeinschaft (DFG, German Research Foundation) Projektnummer 324392634—TRR 221 project B12 (KP and MK).

## Conflict of Interest

The authors declare that the research was conducted in the absence of any commercial or financial relationships that could be construed as a potential conflict of interest.

## Publisher’s Note

All claims expressed in this article are solely those of the authors and do not necessarily represent those of their affiliated organizations, or those of the publisher, the editors and the reviewers. Any product that may be evaluated in this article, or claim that may be made by its manufacturer, is not guaranteed or endorsed by the publisher.
